# Root growth in light of changing magnesium distribution and transport between source and sink tissues in potato (*Solanum tuberosum* L.)

**DOI:** 10.1038/s41598-020-65896-z

**Published:** 2020-05-29

**Authors:** Mirjam Koch, Merle Katharina Winkelmann, Mario Hasler, Elke Pawelzik, Marcel Naumann

**Affiliations:** 1 Department of Crop Sciences, Division Quality of Plant Products, Carl-Sprengel-Weg 1, 37075 Göttingen, University of Göttingen, Göttingen, Germany; 2 Variationsstatistik, 24098 Kiel, Christian-Albrechts-University of Kiel, Kiel, Germany; 3 Present Address: Albrecht Daniel Thaer-Institute of Agricultural and Horticultural Sciences, Crop Science, Albrecht-Thaer-Weg 5, Humboldt-University of Berlin, Berlin, Germany; 4 Present Address: Department of Agricultural Sciences and Landscape Architecture, Albrechtstraße 30, 49076 Osnabrück, University of Osnabrück, Osnabrück, Germany

**Keywords:** Plant sciences, Plant stress responses, Abiotic

## Abstract

This study depicts relations between magnesium (Mg) transport and re-translocation, photoassimilate partitioning, cation and ion concentrations, and finally root growth of potato under different Mg supplies. Potato plants were grown in a hydroponic culture system under different Mg regimes while investigating Mg concentrations, the expression of various Mg transporters, soluble sugars, and cations and anions in source and sink organs at different growth stages. Reports from literature about the impact of Mg deficiency on root growth are inconsistent. As Mg is known to be a phloem mobile nutrient, it is expected to be re-translocated under restricted availability of Mg from source to sink organs. Thus, we assume that plants can tolerate a slight Mg restriction without severe root growth reduction. However, under severe Mg deficiency, the process of Mg re-translocation is hampered, resulting in an impaired photoassimilate partitioning, and finally root growth. This might also explain the findings of studies claiming that Mg deficiency does not impair root growth as plants of these studies likely only suffered a slight Mg restriction. Finally, this study gives indications that an interruption of the process of Mg-re-translocation in early plant growth could be an indicator for growth reductions of the plant at a later growth stage.

## Introduction

Root morphological characteristics, such as root length, diameter, and number, determine a plant’s nutrient efficiency to a great extent^1^. Potato plants are known to have a root system that is shallow and less extended, and are classified as having a poor rooting efficiency^2^. This might contribute to the fact that the potato can be classified as inefficient in the acquisition of nutrients^2,3^. On the other hand, several studies have demonstrated a positive relationship between a sufficient plant mineral status and root growth^4–6^. However, studies on the impact of mineral nutrition on root growth in the potato are rare. Besides, the existing literature on the impact of magnesium (Mg) on root growth seems to be contradictory, at least at first glance. Hermans and Verbruggen^7^ stated that Mg deficiency does not markedly reduce the root development of *Arabidopsis thaliana* plants. In the same plant species and under Mg deficiency, Niu, *et al*.^8^ found increased root hair development and Niu, *et al*.^9^ even increased root biomasses although the formation of root hairs has been discussed to be a strategy by the plant to adapt to changing nutrient availabilities^5^.

Against the findings by Hermans and Verbruggen^7^, Niu, *et al*.^8^ and Niu, *et al*.^9^, Gruber, *et al*.^4^ showed significant root growth reductions in Mg-deficient *Arabidopsis thaliana*, as did Koch, *et al*.^10^ in potato. Besides, Cakmak, *et al*.^11^, Fischer and Bremer^12^, and Neuhaus, *et al*.^13^ found clear root growth reductions in bean plants (*Vicia faba* L.). We assume that these contradictory statements are mainly related to differences in the experimental set-ups of these studies. Plants in the studies conducted by Hermans and Verbruggen^7^, Niu, *et al*.^8^ and by Niu, *et al*.^9^ were already five weeks old before the actual Mg treatments started. In contrast, plants used in the experiment by Gruber, *et al*.^4^ and by Cakmak, *et al*.^11^ were only four to six days old before the Mg treatment started. Plants used in the study by Fischer and Bremer^12^ were 21 days old but the authors removed Mg completely from the nutrient solution when starting the treatment; all the other studies just reduced the Mg concentration in the nutrient solution. The potato plants in the study by Koch, *et al*.^10^ suffered from a deficient Mg supply starting from the first day of growth. Finally, it can be proposed that plants used in the studies of Hermans and Verbruggen^7^, Niu, *et al*.^8^, and Niu, *et al*.^9^ were able to take up adequate Mg prior to the onset of the treatment, which was sufficient for proper root development. Still, it seems to be unclear which Mg supply or Mg tissue concentration is accompanied by an impaired root growth.

An accumulation of soluble sugars in source leaves has been reported to be one of the first symptoms under Mg deficiency caused by a restricted photoassimilate export. This has been shown, for instance, in sugar beet (*Beta vulgaris* L. cv. Adonis)^14^ and in potato (*Solanum tuberosum* L.)^10^. Here, Mg interacts with H^+^-ATPases, which balance charges and provide energy for the phloem loading process.

Owing to the indispensable functions of Mg in plant development, intracellular concentrations of Mg^2+^ are precisely regulated by finely-tuned cellular influx and efflux mechanisms. However, from a molecular point of view, these Mg transport systems are still poorly understood, and most knowledge originates from studies with yeast, *Salmonella*, purified plant mitochondria, and *Arabidopsis thaliana*^15–17^. The transport mechanisms of loading and unloading in vascular tissues, in particular, remain unknown^16^. In prokaryotes, the so-called CorA channel, and in eukaryotes, its homologue *Mitochondrial RNA Splicing 2* (*MRS2*), are the major Mg^2+^ uptake systems^18^. The best-studied Mg transport family identified in higher plants belongs to homologues of the CorA Mg transporters and are called MGTs, standing for magnesium transporters. They exhibit a high similarity with the MRS2 transporters identified in eukaryotes. Therefore, they are known as MGTs as well as MRS2s^19^. In *Arabidopsis*, at least 10 members of these families were identified. However, there is a lack of knowledge as to whether all the members of this transport family actually exhibit Mg transport action^19^. To the best of our knowledge, rice (*Oryza sativa* L.)^20^, rapeseed (*Brassica napus* L.)^21^, maize (*Zea mays* L.)^22^, and soybean (*Glycine max* (L.) Merr.)^20^ are the only commonly used agricultural species where Mg transport systems have been investigated. This indicates a necessity for more research.

The present study was split into Experiments 1 and 2. Experiment 1 intended to validate the findings by Koch, *et al*.^10^ that Mg deficiency results in a restricted root growth in potato and to determine the Mg tissue concentration that is accompanied by these findings. Shifts in the concentration and distribution of Mg within the plant might also severely affect the concentration and distribution of other mineral nutrients and ions. The distribution of nutrients follows distinctive functions^23^, such as the maintenance of cation‒anion balances^24^ or the exhibition of signal function^25^. These specific functions might be interrupted by changing Mg concentrations and distributions. Magnesium is known to be a phloem-mobile nutrient and it is assumed that phloem-mobile mineral nutrients are readily translocated within the plant^26^. Therefore, under restriction of Mg, deficiency symptoms are usually expected to occur first in older tissue while younger tissue is still sufficiently supplied with Mg as it is re-translocated from the older (source) to the younger (sink) tissue via the phloem^27^. Therefore, Experiment 2 focused on root growth in the light of changes in the Mg distribution between source and sink tissue. We assume that root growth reduction is a very late response in plants to Mg deficiency as under a slight Mg restriction, Mg can be re-translocated between plant organs and, so, the plant is still able to maintain Mg-dependent processes without severe growth reduction. One aim of Experiment 2 is to prove this assumption. In order to further characterize Mg distribution and transport processes in potato, the gene expressions of various Mg transporters in leaves in the light of different Mg supplies were investigated.

## Materials and methods

### Experimental design and growth conditions

In Experiment 1, potato plants (*Solanum tuberosum* L.) of the variety ‘Laura’ were grown in a nutrient solution in a climate chamber from May to July in 2016. The plants grew in an alternating day/night cycle of 12 hours and a photosynthetic photon flux density of 180 µmol m^−2^ s^−1^ (MASTER Agro 400 W; Philips, the Netherlands) during illumination. Average air temperature was 20 °C during the day and 16 °C during the dark period and relative humidity was 60%. In Experiment 2, plants of ‘Laura’ were grown in a nutrient solution in an open outdoor installation from August to November in 2017 (mean temperature, precipitation, and irradiance are shown in Supplementary Figure 1). Before the onset of the experiments, plants were propagated in nutrient-poor soil (soil features are shown in Supplementary Table 1). In Experiment 1, the soil was additionally fertilized, as shown in Supplementary Table 2. For planting, tuber pieces with a germ bud were used to avoid nutrient delivery from the whole tuber. When the plants reached a height of approximately 10 cm, they were transferred to a nutrient solution. The nutrient concentrations of the solution were increased stepwise (Experiment 1: 20%–50%–100%; Experiment 2: 20%–40%–60%–100%) over the first seven (Experiment 1) and five days (Experiment 2) respectively. The full nutrient solution concentrations of both experiments were: 1250 µM Ca(NO_3_)_2_ • 4 H_2_O_,_ 250 µM NH_4_NO_3_, 1000 µM K_2_SO_4_, 130 µM Ca(H_2_PO_4_)_2_ • H_2_O, 100 µM Fe(III) EDTA (13% Fe), 10 µM H_3_BO_3_, 1 µM ZnSO_4_ • 7 H_2_O, 1 µM MnSO_4_ • H_2_O, 0.2 µM CuSO_4_ • 5 H_2_O, and 0.14 µM H_24_Mo_7_N_6_O_24_ • 4 H_2_O. Furthermore, the plants were treated with three different Mg supplies: A low (5 µM Mg), a medium (100 µM Mg), and a high supply (500 µM Mg), following designations like ‘Mg low’, ‘Mg med’, and ‘Mg high’ in Experiment 1. In Experiment 2, the plants were treated with two different Mg supplies: A low (5 µM Mg) and a medium supply (100 µM Mg); likewise, they were designated as ‘Mg low’ and ‘Mg med’. Mg was given as MgSO_4_ • 7 H_2_O. The onset of the treatment for both experiments was the day on which the nutrient concentration of the solution reached 100%.

The schematic experimental setups of Experiments 1 and 2 are shown in Supplementary Figure 2. The plants were grown in five-litre plastic pots with one plant per pot. In Experiment 1, each treatment was represented by four plants. In Experiment 2, ‘Mg low’ plants had 19 biological replications and ‘Mg med’ plants had 11 biological replications. The nutrient solution was aerated and changed twice per week, depending on plant growth and water consumption.

### Leaf sampling

**Figure 1:**
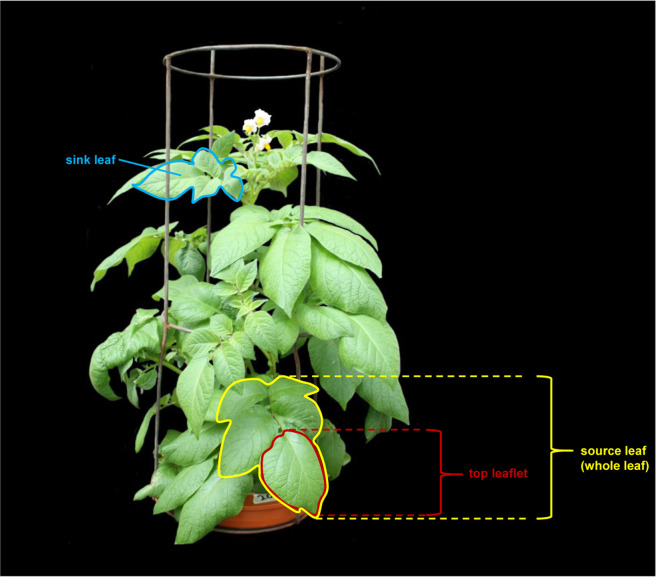
Scheme showing which leaf was treated as sink and which leaf was treated as source leaf and illustrating top leaflet and whole leaf for leaf sampling. In Experiment, 1 only older source leaves, and in Experiment 2, older source and younger sink leaves were sampled. Mineral nutrients were assessed solely in whole leaves. All other analyses were determined in top leaflets.

For Experiment 1, only older, fully developed source leaves were sampled and used for mineral nutrient and soluble sugar determination. For mineral nutrient analyses, solely whole leaflets were taken, while for sugar determination in leaves, only top leaflets were sampled (Fig. 1). In Experiment 2, mineral nutrients, anions, soluble sugars, and the gene expression of Mg transporters were assessed in younger sink leaves, referred to as ‘sink leaves’ in the following, and, additionally, in older source leaves, referred to as ‘source leaves’ in the following (Fig. 1). While mineral nutrients were assessed in whole leaves, for analyses of sugars and of the gene expression of Mg transporters in leaves, only top leaflets were taken (Fig. 1). With respect to the aboveground biomass of potato it is difficult to make a generally valid division into younger (sink) and older (source) parts, as there are emerging new leaves in each section of the plant. Finally, differently as it is for example the case in grain plants where older leaves usually are located in the lower part and younger leaves in the upper part of the plant, in potato older as well as younger leaves can be found in each section of the plant, especially at later growth stages. Still, in the present study, older fully developed leaves were mainly located in the lower part, while younger still developing leaves were rather located in the upper part of the plant (Fig. 1). However, the final decision which leaf was sink and which leaf was source leaf was especially based on characteristics of the leaf itself: Sink leaves were smaller, still growing, and usually lighter green while source leaves were larger, fully developed, and usually darker green.

### Mineral nutrient determination in leaves and roots

For both experiments, the harvested leaf and root samples were dried at 60 °C for four days. Later, leaves were ground with a mortar and pestle, and root samples were ground into 0.5 mm flour in a hammer mill (DFH 48, Culatti, Switzerland). Roots were washed prior to drying to get rid of nutrient residues on the root surface. In Experiment 1 and 2, the minerals (leaves and roots) were determined as described by Koch, *et al*.^10^. However, in Experiment 2 the concentrations of Mg were examined using atom adsorption spectroscopy (AAS Unicam M Solaar, Germany).

### Phenotype, shoot and root growth, and root scanning

The phenotype was documented by taking photographs of representative leaflets and roots. Morphological changes in the shoot were recorded by measuring the plant height with a common tapeline and counting the internodes per plant on a weekly basis. At harvest (55 days after the onset of treatment in Experiment 1 and 78 days after the onset of the treatment in Experiment 2), the complete shoots were cut off and the total shoot biomasses were assessed. Roots were separated from the shoots and stored at −20 °C until root scan analysis. Immediately prior to root scanning, the roots were thawed, stolons were discarded, and the roots were completely covered with water. The further procedure of root scanning was done as described by Koch, *et al*.^10^.

Besides, the total root biomasses were examined, and, based on the dry weights of shoots and roots, the shoot-to-root ratios were determined. The dry weights of roots and shoots were determined by taking the weight of roots and shoots after drying them in a drying chamber at 105 °C for two days.

### Determination of anion contents

20 mg dried and homogenized material was dissolved in 1 ml of 0.1 M HCl. The samples were centrifuged at 23 °C and 25,000 g for 30 min. The supernatant was transferred to a centrifugal filter (PES, MWCO 3kD, VWR, Pennsylvania, USA) and centrifuged at 8,000 g and 23 °C for 60 min. After filtration, the samples were diluted 20 times with deionized water and analysed using the Metrohm ECO IC system, with a Na_2_CO_3_/NaHCO_3_ gradient over a Metrosep A Supp 17 150/4.0 column with a flow of 0.7 mL/min.

### Soluble sugar determination in fully expanded leaves

For Experiment 1, the soluble sugars were examined with the help of different assays for the determination of metabolites. The leaves were shock-frozen in liquid nitrogen immediately after harvest and stored at −80 °C until further analysis. The following quantification of sugars was performed, as described by Koch, *et al*.^10^.

In Experiment 2, soluble sugars were quantified by high-performance liquid chromatography (HPLC) as described by Koch, *et al*.^10^ for the determination of soluble sugars in tubers. Instead of 0.4 gram of potato tuber flour 0.4 gram of potato leaf flour was used. The same flour was used as taken for mineral nutrient analyses.

### RNA extraction and quantitative real-time polymerase chain reaction

RNA extraction was done in accordance with Koch, *et al*.^10^. All primers used for qRT-PCR are listed in Supplementary Table 3 with the corresponding gene names, NCBI Reference Sequences, and UniProtKB names. For qRT-PCR, the same thermal cycling protocol as in Koch, *et al*.^10^ was used. The relative gene expression was evaluated with the Bio-Rad CFX Manager (Bio-Rad Laboratories Inc., California, USA) using *StUbiquitin* as the reference gene.

### Statistics

The statistical analysis was performed using R software Version 3.5.2^28^. Based on a graphical residual analysis, the data were assumed to be normally distributed and heteroscedastic. Depending on the specific measurement variables, statistical mixed models^29,30^ or models based on generalized least squares^31,32^ were used with appropriate fixed and random influence factors. ANOVA and multiple contrast tests^33,34^ were performed in order to detect differences between treatments. The specific R code can be obtained on request from the corresponding author.

## Results

As a reminder, this study was split into Experiments 1 and 2. Experiment 1 mainly intended to validate that Mg deficiency results in a restricted root growth in potato and to determine the Mg tissue concentrations that are accompanied by a restricted root growth. Experiment 2 focused on root growth in the light of changes in the Mg distribution between source and sink tissue in dependence on the respective Mg supply in order to show relations here if present. Besides, Experiment 2 aimed to further characterize how different Mg supplies affect the concentration and distribution of not only Mg but also of other cations and of ions. And secondly, Experiment 2 aimed to show how different Mg supplies affect Mg transport processes. Thus, the gene expressions of various Mg transporters in leaves were investigated under different Mg supplies.

### Leaf magnesium status and corresponding phenotypes of the respective leaves

**Figure 2:**
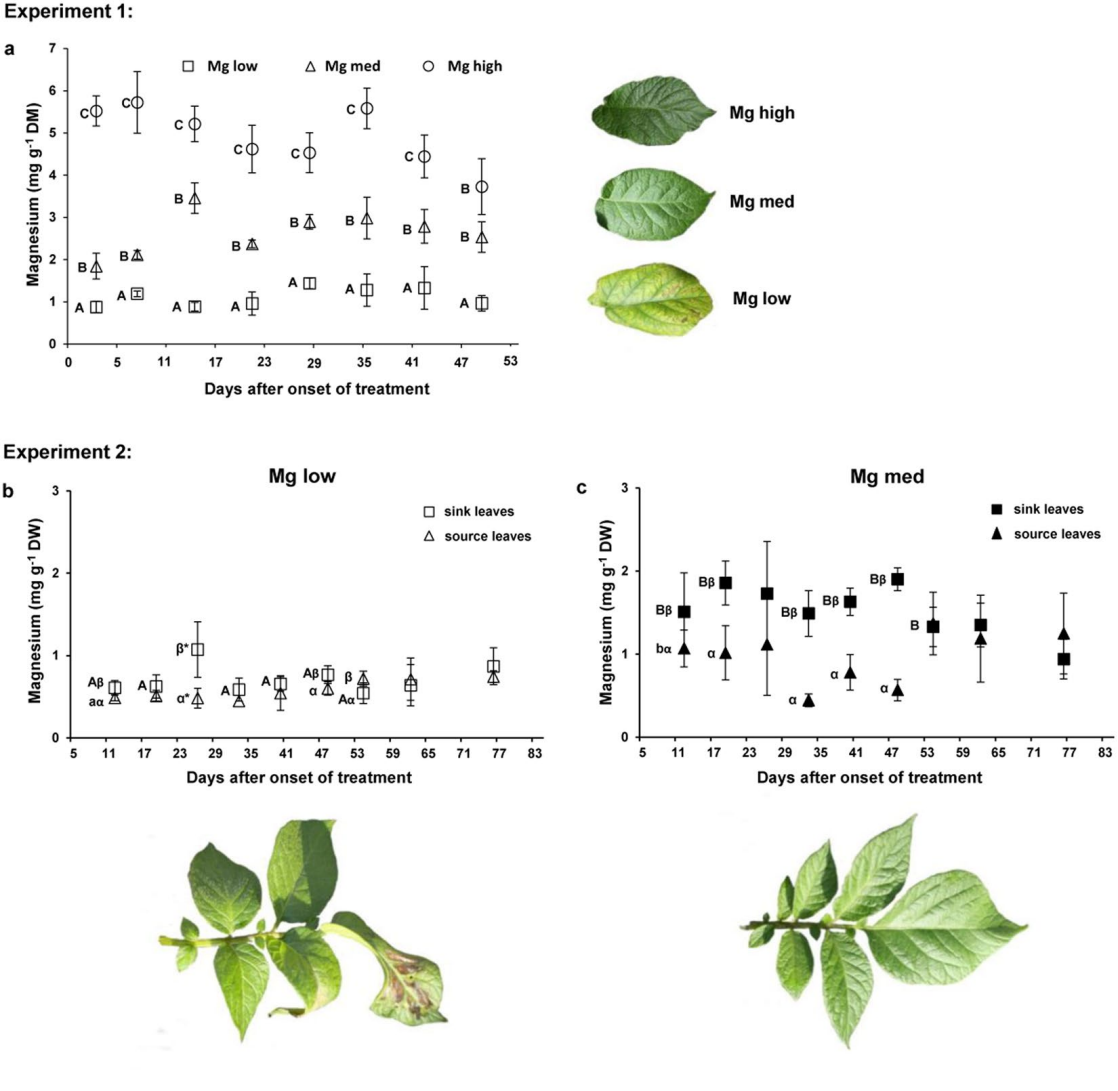
Effect of Mg supply on the Mg leaf status and the corresponding phenotype of the leaves in Experiment 1 (**a**) and 2 (**b and c**). (**a**) Mg leaf concentrations in source leaves of Experiment 1 at eight sampling dates and with representative leaflets (53 days after the onset of the treatment) under low (‘Mg low’), medium (‘Mg med’) and high (‘Mg high’) Mg supply (n = 4). Mg leaf concentrations in sink and source leaves in Experiment 2 at nine sampling dates with representative phenotypes of a sink leaf (55 days after the onset of the treatment) under (**b**) low (‘Mg low’) and (**c**) medium (‘Mg med’) Mg supply (n = 1–4). ‘Mg low’ = 5 µM Mg; ‘Mg med’ = 100 µM Mg; ‘Mg high’ = 500 µM Mg. Mean ± SE values. Capitals = significant differences between ‘Mg low’, ‘Mg med’ and ‘Mg high’ treated plants (Experiment 1) or between ‘Mg low’ and ‘Mg med’ treated plants (Experiment 2) at one sampling date. Small letters = significant differences between ‘Mg low’ and ‘Mg med’ treated plants in source leaves at one sampling date. Greek letters = significant differences between sink and source leaves of ‘Mg low’ or ‘Mg med’ treated plants, respectively at one sampling date. No indication = not significant. *P* < 0.05; * = *P* < 0.01.

In Experiment 1, the Mg leaf concentrations revealed significant differences between the different Mg treatments (Fig. 2a). ‘Mg low’ plants exhibited the significant lowest Mg leaf concentrations with less than 1.4 mg g^−1^ dry weight (DW) on all sampling dates (Fig. 2a). ‘Mg med’ plants always showed higher Mg leaf concentrations than 2 mg g^−1^ DW with the exception of the first sampling date when the Mg concentration was below 2 mg g^−1^ DW. ‘Mg high’ plants exhibited throughout all sampling dates at least fourfold higher Mg concentrations compared to ‘Mg low’ plants. The different Mg leaf concentrations are also illustrated by the different representative older leaflets of ‘Mg low’, ‘Mg med’, and ‘Mg high’ plants, of which ‘Mg low’ plants showed clear chloroses in between the leaf veins and necroses starting near the tip of the leaf (Fig. 2a). Meanwhile, the leaves of ‘Mg med’ and ‘Mg high’ plants did not show these symptoms and appeared lush green.

Likewise as in Experiment 1, the Mg leaf concentrations of ‘Mg low’ plants were significantly lower compared to ‘Mg med’ plants in Experiment 2 (Fig. 2b,c). This was especially pronounced in sink leaves (Fig. 2b,c). However, the Mg concentrations did not show significant deviations on the last two sampling dates. In ‘Mg low’ plants, the Mg leaf concentrations were clearly below 1 mg g^−1^ DW in sink as well as in source leaves at all sampling dates, except in sink leaves at the third sampling date. Here, the average Mg leaf concentrations were slightly higher than 1 mg g^−1^ DW. Besides, the Mg leaf concentrations of ‘Mg low’ plants showed less deviation between sink and source leaves compared to ‘Mg med’ plants. ‘Mg med’ plants revealed more distinct differences in their Mg leaf concentrations between sink and source leaves with the exception of the last sampling date (Fig. 2c). While source leaves demonstrated Mg leaf concentrations of around 1 mg g^−1^ DW and slightly lower, sink leaves revealed Mg leaf concentrations of around 1.5‒2 mg g^−1^ DW, with the exception of the last sampling date, where the Mg leaf concentrations accounted for, on average, 1 mg g^−1^ DW. The lower Mg leaf concentrations of ‘Mg low’ plants compared to ‘Mg med’ plants, especially in sink leaves, are also illustrated in the phenotypes of a sink leaf of a representative ‘Mg low’ (Fig. 2a) and a ‘Mg med’ plant (Fig. 2b) at 55 DAO. The leaf of the ‘Mg low’ plant shows clear chloroses and necroses, especially on the front leaflet, while the leaf of the ‘Mg med’ plant does not show any symptom of chloroses and necroses.

### Root and shoot growth

**Figure 3:**
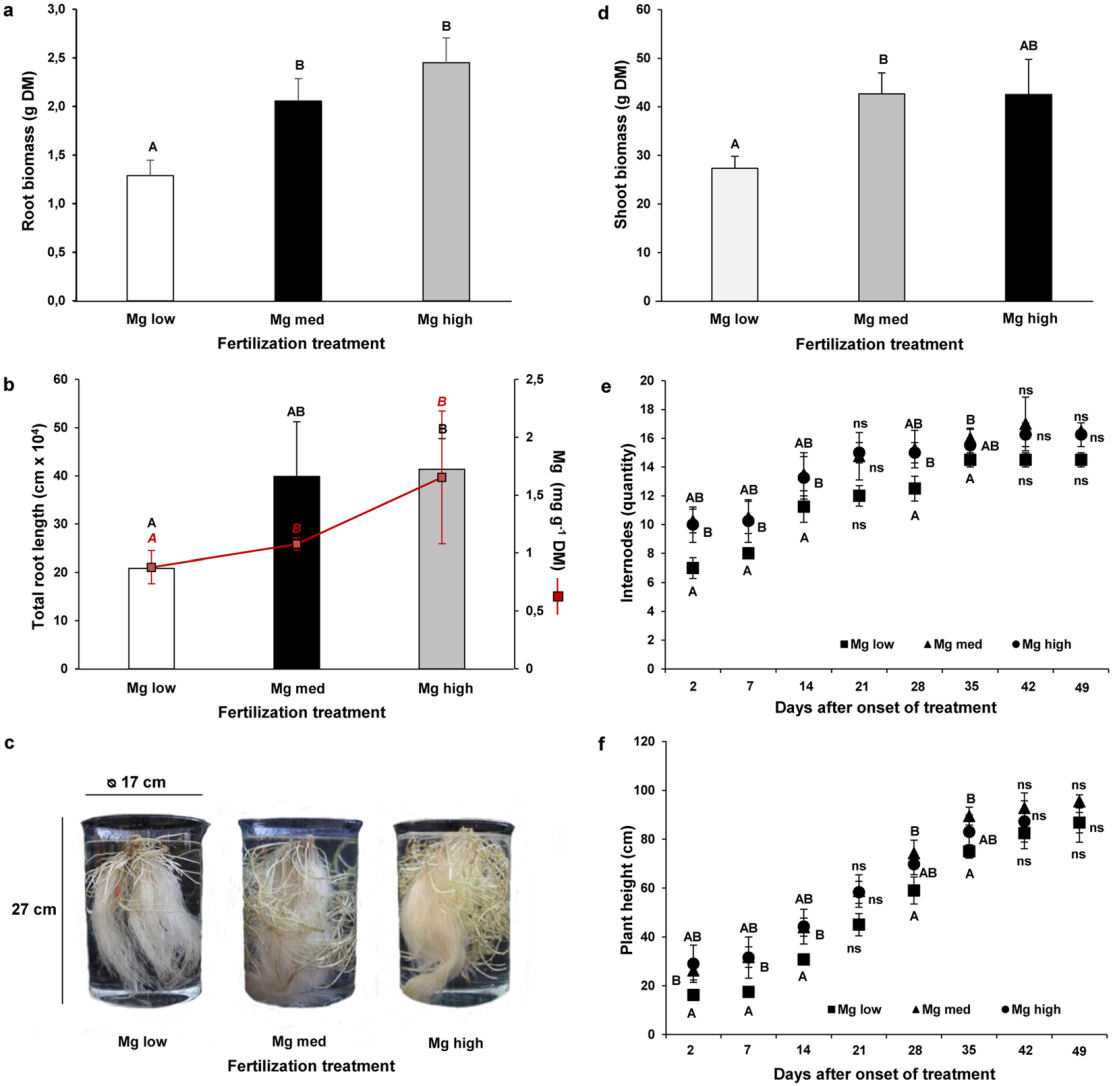
Effect of low, medium, and high Mg supply on the below- and aboveground biomass (Experiment 1). (**a**) Root biomass, (**b**), total root length, and (**c**) phenotypes of potato roots, stolons, and mini tubers in a beaker of 5 liter volume under low (‘Mg low’), medium (‘Mg med’), and high (‘Mg high’) Mg supply at harvest (55 days after the onset of the treatment (n = 4). (**d**) Shoot biomass at harvest (55 days after the onset of the treatment), (**e**) number of internodes, and (**f**) plant heights under low (‘Mg low’), medium (‘Mg med’), and high (‘Mg high’) Mg supply (n = 4). ‘Mg low’ = 5 µM Mg; ‘Mg med’ = 100 µM Mg; ‘Mg high’ = 500 µM Mg. Mean ± SE values. Capitals = significant differences between ‘Mg low’, ‘Mg med’, and ‘Mg high’ treated plants. No indication = not significant. *P* < 0.05; * = *P* < 0.01.

Plants of Experiment 1 demonstrated a significant reduction in root biomass in ‘Mg low’ compared to ‘Mg med’ and ‘Mg high’ plants: ‘Mg low’ plants exhibited more than 40% less root biomass compared to the higher Mg fertilization treatments (Fig. 3a). However, ‘Mg med’ and ‘Mg high’ plants did not show significant differences in the root biomasses (Fig. 3a). This also becomes evident in the representative photographs of plant roots of each Mg treatment (Fig. 3c). While roots of ‘Mg med’ and ‘Mg high’ plants appear longer and denser and lie on the bottom of the beaker, roots of ‘Mg low’ plants are shorter and appear much thinner and almost do not reach the bottom. A similar pattern emerged in the total root length of the differently Mg-treated plants: ‘Mg low’ plants showed, on an average, more than a half reduction in total root length compared to ‘Mg med’ and ‘Mg high’ plants (Fig. 3b). Moreover, the Mg root concentrations were significantly reduced in ‘Mg low’ plants compared to ‘Mg med’ and ‘Mg high’ plants (Fig. 3b). Similar results for root biomass, total root length, and root Mg concentrations were assessed in Experiment 2 between ‘Mg low’ and ‘Mg med’ plants (Supplementary Figure 3b,c).

Also the shoot growth demonstrated significant reductions in ‘Mg low’ plants in Experiment 1 (Fig. 3d). However, Mg deficiency leads to a more severe reduction in root biomass (Fig. 3a) compared to shoot biomass (Fig. 3a). This was reflected in higher shoot-to-root biomass ratios of ‘Mg low’ plants compared to ‘Mg med’ and ‘Mg high’ plants: ‘Mg low’ plants exhibited, on an average, a shoot-to-root ratio of 22 ± 3, while the shoot-to-root ratio of ‘Mg med’ plants was, on an average, 21 ± 2, and of ‘Mg high’ plants 17 ± 3. Similar results were found in Experiment 2 (Supplementary Figure 3a,b). Besides, ‘Mg low’ plants exhibited the lowest number of internodes (Fig. 3e) and the lowest plant heights in Experiment 1 (Fig. 3f). However, on the last two sampling dates, the number of internodes and the plant height did not show any significant differences between the different Mg treatments. In Experiment 2, the plant height did not even show significant differences at all between ‘Mg low’ and ‘Mg med’ plants (Supplementary Figure 4a). Only the number of internodes was significantly reduced in ‘Mg low’ plants from the fourth sampling date onwards (Supplementary Figure 4b).

### Mineral nutrient concentrations others than Mg in source leaves and roots

Potassium leaf concentrations were significantly higher in ‘Mg low’ compared to ‘Mg high’ plants in Experiment 1 (Table 1). Besides, the micronutrients manganese (Mn) and sodium (Na) exhibited, significantly, the highest concentrations in leaves of ‘Mg low’ plants. Significantly, the lowest leaf concentrations were revealed for the macronutrient sulphur (S) and the micronutrient iron (Fe) in ‘Mg low’ compared to ‘Mg med’ plants.

**Table 1:** Mineral nutrient concentrations in mg g^−1^ DW in source leaves at 49 days after onset of the treatment (three days before root harvest) and in roots at harvest (55 days after onset of the treatment) (n = 4) (Experiment 1).

Leaves						
	Mg low		Mg med		Mg high	
K	66.88 ± 2.63	B	60.71 ± 5.38	AB	56.65 ± 4.44	A
Ca	12.32 ± 3.16		12.31 ± 1.03		14.04 ± 3.43	
P	6.12 ± 1.34		6.75 ± 1.63		4.79 ± 1.38	
S	3.13 ± 0.40	A	4.10 ± 0.52	B	3.52 ± 0.84	AB
B	0.05 ± 0.01		0.05 ± 0.01		0.05 ± 0.01	
Fe	0.10 ± 0.01	A	0.16 ± 0.03	B	0.11 ± 0.02	AB
Mn	0.06 ± 0.01	B	0.04 ± 0.01	A	0.05 ± 0.01	AB
Na	0.19 ± 0.04	B	0.09 ± 0.02	A	0.06 ± 0.02	A
Zn	0.06 ± 0.02		0.05 ± 0.02		0.03 ± 0.01	
**Roots**						
K	2.32 ± 1.27		1.71 ± 0.22		3.86 ± 2.58	
Ca	13.83 ± 2.27	AB	13.91 ± 1.18	B	11.33 ± 0.46	A
P	4.46 ± 0.94		3.86 ± 0.32		3.47 ± 1.09	
S	2.87 ± 0.52		3.11 ± 0.16		3.29 ± 0.32	
B	0.02 ± 0.01		0.02 ± 0.01		0.02 ± 0.01	
Fe	4.58 ± 0.41	B	2.36 ± 0.61	A	1.98 ± 0.86	A
Mn	0.33 ± 0.07	B	0.17 ± 0.02	A	0.14 ± 0.05	A
Na	0.35 ± 0.02		0.36 ± 0.03		0.37 ± 0.03	
Zn	0.18 ± 0.07	B	0.04 ± 0.01	A	0.04 ± 0.01	A

‘Mg low’ = 5 µM Mg; ‘Mg med’ = 100 µM Mg; ‘Mg high’ = 500 µM Mg. K = potassium, Ca = calcium, P = phosphorus, S = sulphur, B = boron, Fe = iron, Mn = manganese, Na = sodium, Zn = zinc. Mean ± SE values. Capitals = significant differences between ‘Mg low’, ‘Mg med’, and ‘Mg high’ plants. No indication = not significant. *P* < 0.05.

The macronutrient concentrations in roots showed no significant differences between the different Mg supplied plants with the exception of calcium (Ca): ‘Mg med’ plants exhibited significantly higher Ca concentrations compared to ‘Mg high’ plants (Table 1). The Mn concentrations showed a similar pattern in roots as in leaves: The ‘Mg low’ plants showed significantly higher Mn concentrations compared to ‘Mg med’ and ‘Mg high’ plants. However, the Fe concentrations demonstrated a contrary pattern in the roots compared to the leaves: The root Fe concentrations were, significantly, the highest in ‘Mg low’ plants compared to ‘Mg med’ and ‘Mg high’ plants. Finally, the root zinc (Zn) concentrations were significantly higher in ‘Mg low’ plants compared to ‘Mg med’ and ‘Mg high’ plants.

### Ion concentrations in sink and source leaves

**Figure 4:**
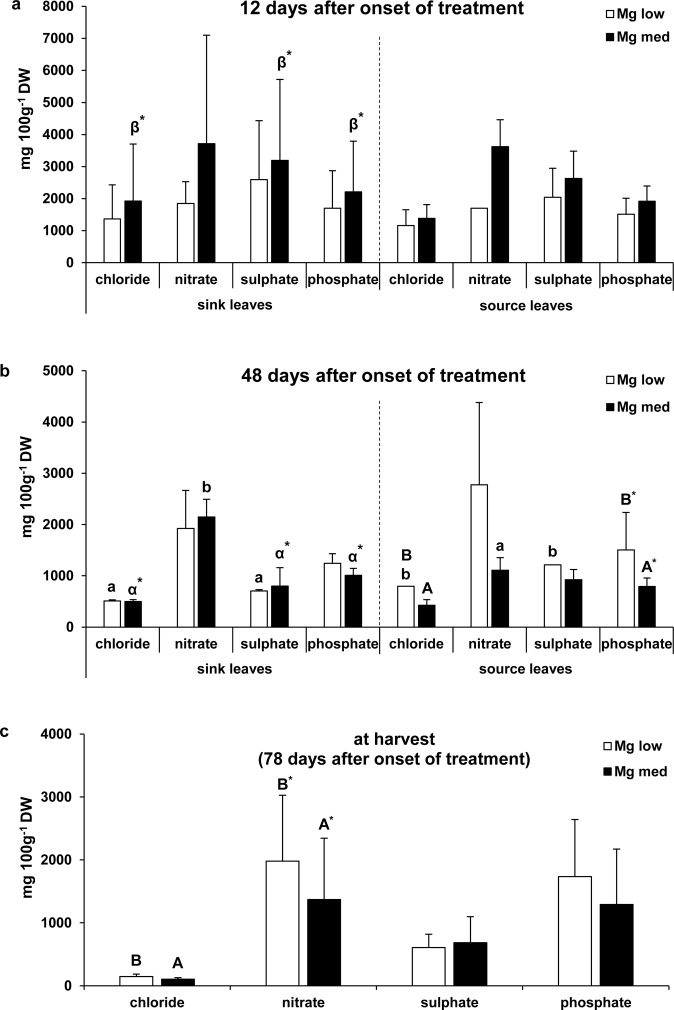
Chloride, nitrate, sulphate, and phosphate concentrations under low and medium Mg supply (Experiment 2). Ion concentrations in sink (left) and source (right) leaves under low (‘Mg low’) and medium (‘Mg med’) Mg supply (**a**) 12 and (**b**) 48 days after the onset of the treatment (n = 1–4). (**c**) Ion concentrations in the shoots (stems and leaves) under low (‘Mg low’) and medium (‘Mg med’) Mg supply at harvest (n = 11–14). ‘Mg low’ = 5 µM Mg; ‘Mg med’ = 100 µM Mg. Mean ± SE values. Capitals = significant differences between ‘Mg low’ and ‘Mg med’ treated plants in source or sink leaves and at one time point (12 or 48 days after the onset of the treatment or at harvest). Small letters = significant differences between sink and source leaves under one Mg treatment (‘Mg low’ or ‘Mg med’) and at one time point. Greek letters = significant differences between 12 and 48 days after the onset of the treatment in source or sink leaves and under one Mg treatment. No indication = not significant. *P* < 0.05; *P < 0.1.

The chloride, nitrate, sulphate, and phosphate concentrations were higher in ‘Mg med’ compared to ‘Mg low’ plants 12 days after the onset of the treatment (Fig. 4a) without exception. However, these differences were not significant. At 48 days after the onset of the treatment, ‘Mg med’ plants showed decreases in the ion concentrations compared to 12 days after the onset of the treatment, with significant effects especially in sink leaves (Fig. 4a,b). Besides, at 48 days after the onset of the treatment, it was the opposite case in source leaves: Here, ‘Mg low’ plants showed higher ion concentrations compared to ‘Mg med’ plants, with significance for chloride and phosphate (Fig. 4b). This effect was also visible at harvest (78 days after the onset of the treatment). Besides, nitrate revealed significant higher concentrations in ‘Mg low’ compared to ‘Mg med’ plants at harvest (Fig. 4c). Furthermore, at 48 days after the onset of the treatment, significant higher chloride and phosphate concentrations were determined in ‘Mg low’ plants in sink compared to source leaves and significant higher nitrate concentrations in ‘Mg med’ plants in sink compared to source leaves (Fig. 4b).

### Soluble sugar concentrations in sink and source leaves

In Experiment 1, the sum of hexose sugars (glucose and fructose) did not show any significant differences between the different Mg treatments on Day 7 after the onset of the treatment (Table 2). But 28 days after the onset of the treatment, ‘Mg low’ plants showed significantly higher hexose sugar concentrations compared to ‘Mg med’ and ‘Mg high’ plants. Moreover, on the last sampling date, ‘Mg low’ plants exhibited threefold higher hexose sugar concentrations compared to ‘Mg med’ and ‘Mg high’ plants. However, these differences were not significant. In Experiment 2, similar patterns were found in the form of higher hexose sugar concentrations in ‘Mg low’ compared to ‘Mg med’ plants (Supplementary Figure 5), however, without any significant effect. Besides, there were no significant differences in hexose sugar concentrations between sink and source leaves.

**Table 2:** Fructose, glucose and total hexose sugar (sum fructose + glucose) concentrations in source leaves of all fertilization treatments at day 7, 28 and 49 after onset of the treatment (DAO) (n = 2 - 4) (Experiment 1).

	Fructose			Glucose			Sum fructose + glucose		
	Mg low	Mg med	Mg high	Mg low	Mg med	Mg high	Mg low	Mg med	Mg high
7 DAO	5.12 ± 3.96	12.15 ± 5.73	5.55 ± 5.48	10.12 ± 5.75	0.38 ± 1.27	2.35 ± 1.48	15.24 ± 4.68	12.53 ± 7.36	7.12 ± 8.66
28 DAO	21.66 ± 8.44	1.38 ± 0.48	0.06 ± 0.01	2.32 ± 1.58	6.49 ± 1.13	5.04 ± 4.77	23.41 ± 7.87 B	7.53 ± 0.56 A	3.38 ± 5.57 A
49 DAO	20.27 ± 11.43	1.38 ± 1.28	2.51 ± 1.27	10.49 ± 7.42	1.59 ± 1.09	1.24 ± 1.08	30.76 ± 21.53	2.18 ± 2.73	2.18 ± 2.93

‘Mg low’ = 5 µM Mg; ‘Mg med’ = 100 µM Mg; ‘Mg high’ = 500 µM Mg. Mean ± SE values. Capitals = significant differences between ‘Mg low’, ‘Mg med’, and ‘Mg high’ plants. No indication = not significant. *P* < 0.05.

### Relative gene expression of Mg transporters in source and sink leaves at different time points

**Figure 5:**
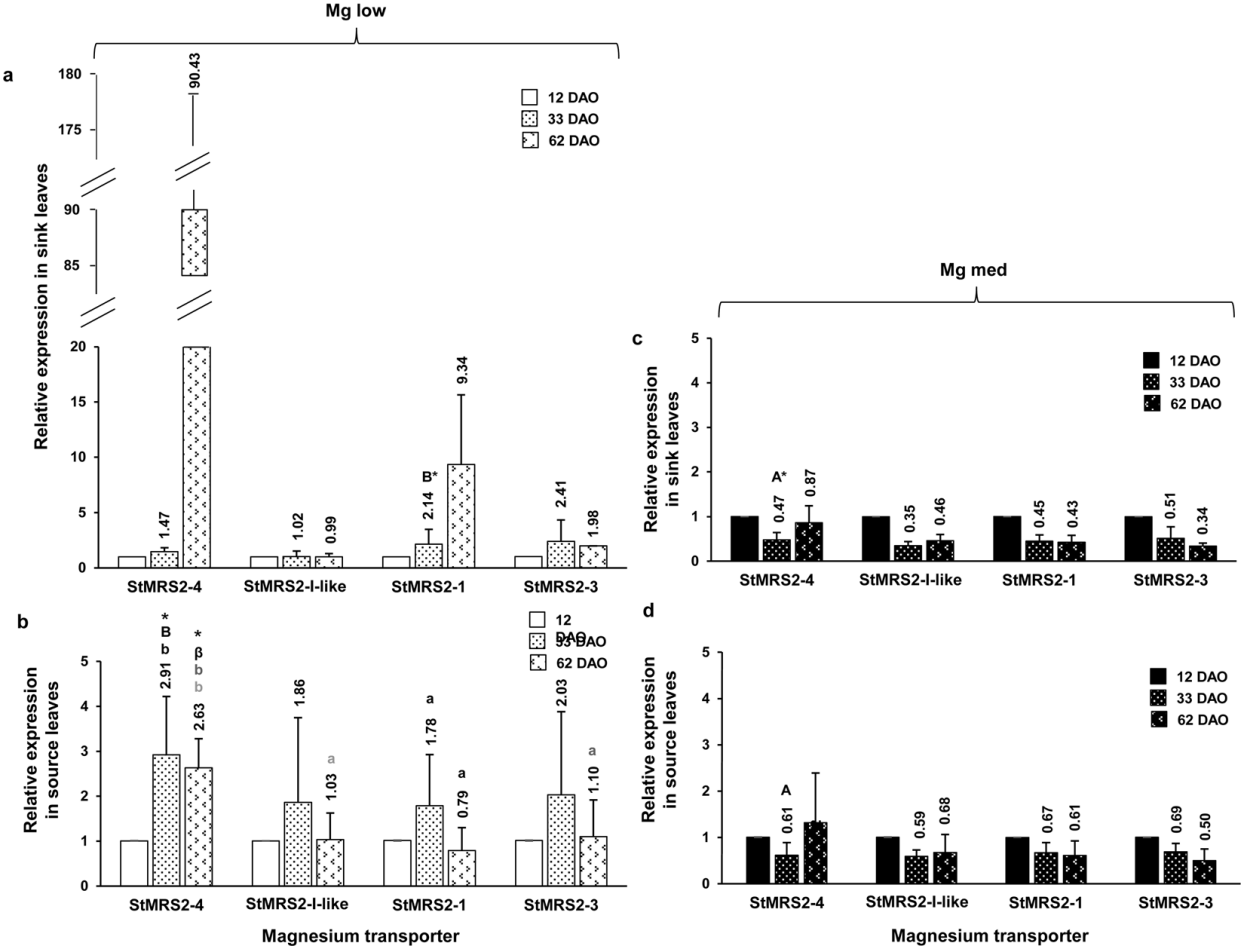
Leaf transcript levels of genes encoding Mg transporters under low and medium Mg supply in sink and source leaves at different sampling dates (Experiment 2). Transcript levels of the Mg transporters *StMRS-4*, *StMRS2-I-like*, *StMRS2-1*, and *StMRS2-3* under low Mg supply (‘Mg low’) in (**a**) sink and (**b**) source leaves and under medium Mg supply (‘Mg med’) in sink (**c**) and (**d**) source leaves at 33 and 62 days after the onset of the treatment compared to 12 days after onset of the treatment (n = 2–4). ‘Mg low’ = 5 µM Mg; ‘Mg med’ = 100 µM Mg. Mean (above the bar plots) ± SE values. Capitals = significant differences between ‘Mg low’ and ‘Mg med’ treated plants of one transporter, of one sampling date, and of sink or source leaves. Small letters = significant differences between transporters of one sampling date, of sink or source leaves, and of one Mg treatment (‘Mg low’ or ‘Mg med’ treated plants). Asterisks = significant differences between sampling dates of one transporter, of sink or source leaves, and of one Mg treatment (‘Mg low’ or ‘Mg med’ treated plants) compared to control plants (12 DAO). No indication = not significant. *P* < 0.05; *P < 0.01.

The relative gene expressions of the Mg transporters investigated were higher in ‘Mg low’ compared to ‘Mg med’ plants (Fig. 5a–d), with significant effects shown by the transporter *StMRS2-1* in sink leaves at 33 DAO and by the transporter *StMRS2-4* in source leaves at 33 and 62 DAO (Fig. 5a,b). In ‘Mg low’ plants, the relative transcript abundance of Mg transporters was, on an average, higher in sink compared to source leaves (Fig. 5a,b), even though there was no significant difference between sink and source leaves. The Mg transporter *StMRS2-4* showed the highest average expression levels especially in the sink leaves of ‘Mg low’ plants with an expression level of 90 at 62 DAO (Fig. 5a). Besides, the transporter *StMRS2-1* showed high expressions with an average relative expression of almost 10 in the sink leaves of ‘Mg low’ plants at 62 DAO (Fig. 5a). In source leaves of ‘Mg low’ plants (Fig. 5b), likewise as in the sink leaves, the transporter *StMRS2-4* exhibited the highest expression levels.

In ‘Mg med’ plants, the relative gene expression of all Mg transporters was always lower than the control (12 DAO), with the exception of the transporter *StMRS2-4* in source leaves at 62 DAO (Fig. 5c,d). However, here, the average relative expression only revealed 1.32 and did not show any significant difference compared to other transporters, sampling dates, sink leaves, or ‘Mg low’ plants (Fig. 5d).

## Discussion

Root growth was significantly inhibited in ‘Mg low’ compared to ‘Mg med’ and ‘Mg high’ plants in this study (Fig. 3a-c; Supplementary Fig. 3b,c). Usually, Mg tissue concentrations of 2 mg g^−1^ DW are considered to be a critical value to ensure a sufficient Mg supply of the plant^35,36^. However, there can be great deviations between different species and probably also between different cultivars. In Experiment 2 of the present study, ‘Mg med’ plants showed twice as much root biomass and three times as much total root length compared to ‘Mg low’ plants (when comparing the mean values) (Supplementary Fig. 3b,c). Besides, ‘Mg med’ plants revealed clear differences in the Mg leaf concentrations between source and sink leaves (Fig. 2c): While source leaves exhibited Mg leaf concentrations ±1 mg g^−1^ DW, sink leaves had Mg leaf concentrations of ±1.5 mg g^−1^ DW. This indicates that Mg still was able to be re-translocated from source to sink organs why these plants, despite the low level of Mg concentrations in leaves, did not yet suffer a Mg deficiency. Therefore, these plants were still able to maintain all growth processes. Meanwhile, in ‘Mg low’ plants the process of Mg re-translocation was interrupted, as both source and sink leaves exhibited similar Mg concentrations (below 1 mg g^−1^ DW) starting already 11 days after the onset of treatment (Fig. 2b). This resulted in a significant reduction of the root and also shoot growth (Supplementary Fig. 3).

Plants of Experiment 1 exhibited slightly higher Mg leaf concentration in source leaves of ‘Mg low’ and ‘Mg med’ plants compared Experiment 2 (Fig. 2). This was probably the case, as the soil used for plant propagation prior to translation into the hydroponic culture system in Experiment 2 was not fertilized contrary to Experiment 1.

Similar findings—in comparison with ours— with respect to the Mg re-translocation were made by Scott and Robson^37^ in wheat (*Triticum aestivum* L.) in consequence of an inadequate Mg supply. Here, the authors found even lower Mg leaf concentrations in young (sink) compared to old (source) leaves. Therefore, the authors suggested that the Mg concentrations of young leaves could be a more reliable indicator of the respective Mg supply of the plant in order to predict possible growth reductions due to a Mg deficiency. If applying this to our findings it can be stated that Mg leaf concentrations of sink leaves lower than 1 mg g^−1^ DW are most likely leading to root and shoot growth reductions. Meanwhile, Mg leaf concentrations around 1.5 mg g^−1^ DW in sink leaves seem to be already enough to ensure a sufficient root growth. Besides, it can be concluded that an interruption of the re-translocation of Mg between sink and source organs starting already early in growth can be an indicator for a deficient Mg status of the plant which likely will result in a reduction of the root and the shoot growth.

The fact that the re-translocation of Mg from the source to the sink tissue was interrupted in ‘Mg low’ plants in the present study is also strengthened by the respective phenotypes of sink leaves of ‘Mg low’ and ‘Mg med’ plants: In ‘Mg low’ plants, sink leaves showed clear Mg deficiency symptoms (Fig. 2b), while this was not the case for sink leaves of ‘Mg med’ plants (Fig. 2c). Besides, ‘Mg low’ plants exhibited significant soluble sugar accumulations in leaves in this study (Table 2; Supplementary Fig. 5). This is likely the result of an increased activity of the enzyme invertase caused by an accumulation of sucrose in these leaves—as has been discussed, for instance, by Koch, *et al*.^10^. Moreover, Experiment 2 could show that soluble sugars accumulated in source as well as in sink leaves in ‘Mg low’ plants (Supplementary Fig. 5), indicating that the export of photoassimilates was restricted in source as well as in sink leaves. Meanwhile, compared to ‘Mg low’ plants, ‘Mg med’ plants revealed much lower soluble sugar concentrations in source as well as in sink leaves, indicating that especially the source leaves were still able to export sucrose. A main reason for the reduced growth processes found in this study is likely the hampered transport of photoassimilates from source to sink organs due to the indispensable role of Mg for loading the phloem with photoassimilates. Both, a restricted photoassimilate export and an impaired root growth have been likewise shown in potato by Koch, *et al*.^10^.

A further consequence of an impaired Mg re-translocation due to Mg deficiency is a changed expression of genes encoding for Mg transporters: Leaves of ‘Mg low’ plants showed increased transcript levels of the Mg transporters investigated, especially of the transporters *StMRS-4* and *StMRS2-1* in sink leaves (Fig. 5a,b). Homologues of the latter transporters have been shown previously in other plant species to show upregulated gene expressions under Mg deficiency. Mao, *et al*.^38^ found an upregulation of *AtMGT6/AtMRS2-4* in roots of *Arabidopsis thaliana* within 12 hours of being transferred to an Mg-deficient medium. Silencing of the *AtMGT6/AtMRS2-4* gene resulted in a plant growth depression and low Mg status of the plant under limitation of Mg. This phenomenon was reversed after a re-supply of Mg. This indicates a role of the *AtMGT6/AtMRS2-4* transporter in the uptake of Mg under low availability of Mg in the plant. Contrary to our findings, they did not find any difference in the expression levels of leaves (only of roots) of differently Mg-supplied plants. However, the exposure of the investigated plants to Mg deficiency lasted only 12 hours. In our findings, *StMRS2-4* showed the highest relative transcript levels in source leaves at 33 DAO (Fig. 5b) and in sink leaves at 62 DAO (Fig. 5a). This observation may indicate that the gene expression of the investigated Mg transporters in the leaves is affected later in the growth stage compared to the gene expression in roots.

Zhang, *et al*.^20^ were able to show that Mg deficiency highly induced the expression of *OsMGT1* in rice shoots; it was a homologue of the *StMRS2-1* transporter. During the first three days, no increase was detectable in the gene expression of *OsMGT1* upon Mg deficiency. However, the gene expression rapidly increased within the following four days of treatment. This also confirms the idea that in case of Mg deficiency, the Mg transport systems in shoots are affected later in the growth stage compared to Mg transport systems in roots. Furthermore, Zhang, *et al*.^20^ were able to find a change in the tissue expression pattern of *OsMGT1*: Under sufficient Mg supply, the authors mainly detected an expression of *OsMGT1* in the phloem region of the vascular bundle in the shoots. However, under Mg deficiency, they accessed an increased expression of *OsMGT1* in the xylem parenchyma and in the mesophyll cells. Zhang, *et al*.^20^ suggested that the plant strives to ensure the uptake of Mg under the restricted availability by enhancing the unloading of Mg from the xylem and the import of Mg into mesophyll cells. We assume that this assumption is also reflected in our findings. The increased expression of the investigated Mg transporters in source leaves—especially at 33 DAO (Fig. 5b)—indicates the efforts of the plant to increase the unloading of Mg from the xylem via the respective transporters. Following this, Mg usually is re-translocated from source to sink tissues via the phloem, especially under a restricted availability of Mg^27,39^. Here, an increased import of Mg into mesophyll cells can be expected under Mg restriction, as suggested by Zhang, *et al*.^20^. However, when comparing Fig. 2b with Fig. 2c, it becomes obvious that this re-translocation was inhibited in ‘Mg low’ plants (Fig. 2b), as discussed above. This probably resulted in an increased expression level of *StMRS2-4* and *StMRS2-1* in sink leaves (Fig. 5a), possibly facilitating the import of Mg into mesophyll cells. However, this did not lead to an increased transport of Mg due to the absence of Mg.

Another observation of a consequence due to Mg deficiency in the present study was a shift of cationic mineral nutrients and ions. ‘Mg low’ plants showed increases in cationic mineral nutrients in shoot and roots. In particular, K and also the micronutrients manganese (Mn) and sodium (Na) were found in significantly higher concentrations in ‘Mg low’ compared to ‘Mg med’ and ‘Mg high’ plants in the shoot (Table 1). In the root, Fe, Mn, and Zn showed significantly higher concentrations in ‘Mg low’ compared to ‘Mg med’ and ‘Mg high’ plants (Table 1). Such increases in the number of other cations under deficiency of one cation have often been reported^40–42^. This might be attributed to the specific uptake mechanism for nutrients, which discriminate the uptake of other nutrients, especially under excess or deficient supply of a certain nutrient^43,44^. Furthermore, plants make efforts to absorb other cations where there is a deficiency of one cation in order to balance the amounts of cations and anions in the plant^45^.

Besides, a severe Mg deficiency, as found in ‘Mg low’ plants of Experiment 2, revealed an alteration in the ion (chloride, nitrate, sulphate, and phosphate) concentrations compared to ‘Mg med’ plants in dependence on growth and leaf age (Fig. 4). Twelve days after the onset of the treatment, ‘Mg med’ plants exhibited much higher ion concentrations compared to ‘Mg low’ plants (Fig. 4a), most likely due to an overall superior development status of ‘Mg med’ compared to ‘Mg low’ plants. Therefore, ‘Mg med’ plants were probably able to absorb more ions and also use them metabolically. The latter assumption is also reflected in a significant decrease of ions 48 compared to 12 days after the onset of the treatment. Owing to a successful metabolic use of the ions, the concentrations of unbound and measurable ions decreased. Meanwhile, ‘Mg low’ plants showed higher ion concentrations compared to ‘Mg med’ plants 48 days after the onset of the treatment, with significance for chloride and phosphate, especially in source leaves (Fig. 4b). At harvest (78 days after the onset of the treatment), ‘Mg low’ plants still exhibited higher ion concentrations compared to ‘Mg med’ plants, with significance for chloride and nitrate (Fig. 4c). Magnesium has been shown to be essential for the nitrogen metabolism as it is first a structural component of the enzyme glutamine synthetase and second has a catalytic role for the activity of this enzyme^46,47^. The glutamine synthetase generates glutamine from glutamate and ammonium and, so, is essential for the first step of ammonium fixation in plants^48^. Thus, under Mg deficiency, nitrogen is less fixated, explaining the increased nitrate concentrations in ‘Mg low’ plants 48 and 78 days after the onset of the treatment (Fig. 4b,c).

The higher chloride concentrations found in ‘Mg low’ plants may be related to the higher nitrate concentrations as both concentrations show very similar patterns in their concentrations (Fig. 4). This assumption is based on the fact that both chloride and nitrate use the same proteins for uptake without selectivity for one ion^49^. It could be conceivable that because of the decrease in ammonium fixation, the plant expects a deficient status of nitrogen, and, therefore, strives to take up more nitrogen. Thereby, also increasing amounts of chloride may be taken up due to the missing selectivity of the respective uptake mechanisms for nitrate and chloride. The potato crop has often been discussed to be a chloride-sensitive crop^50^, which is why increasing chloride concentrations should be regarded with caution. Chloride sensitive plants are reported to tolerate chloride shoot concentrations in the range of 4–7 mg g^−1^ DW while less sensitive crops are supposed to tolerate chloride shoot concentrations in the range of 15–33 mg g^−1^ DW^51,52^. Assuming that potato is classified as chloride-sensitive, the plants in the present study would exhibit already critical chloride tissue concentrations. However, recently, Hütsch, *et al*.^53^ could show that potato plants reached chloride leaf concentrations of 65–74 mg g^−1^ DW while not showing any toxicity symptoms or growth reductions.

**Figure 6:**
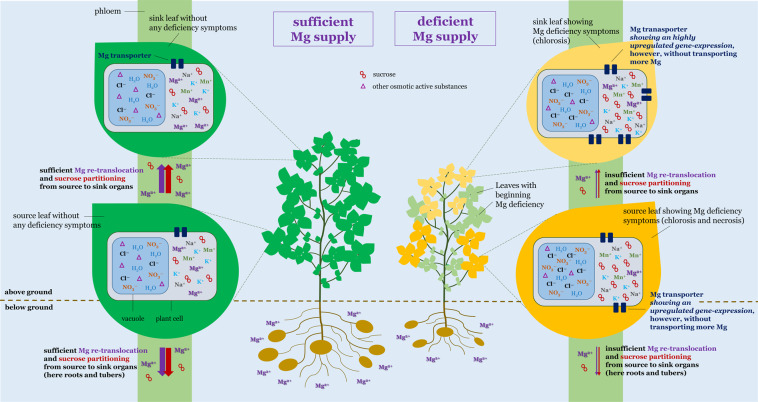
Scheme showing the relations between Mg status, photoassimilate partitioning, Mg transport, concentrations of other cations and of ions, and finally root, shoot, and tuber growth of potato. Magnesium deficiency (right site) initially results in a reduced concentration of Mg in older fully developed source leaves leading in further step in an interruption of the re-translocation of Mg from source to sink organs (younger developing leaves, roots and tubers). The deficient Mg status is getting visible via the development of chloroses and necrosis of source leaves, while sink leaves mainly suffer only chloroses as the older leaves suffer a Mg deficient status already for a longer time period. The deficient Mg status leads to an upregulation of the gene expression of Mg transporters, especially in the sink leaves, however, without transporting more Mg. Besides a hampered re-translocation of Mg, the partitioning of photoassimilates from source to sink organs is restricted. This leads to an accumulation of sucrose in the apoplasm of plant cells. The insufficient re-translocation of Mg and in consequence the impaired partitioning of photoassimilates finally causes a reduced root, shoot, and tuber growth. Furthermore, Mg deficiency is characterized by an increase of other cationic minerals and of ions such as nitrate and chloride, especially in source leaves. Meanwhile, under a sufficient Mg supply (left site) the re-translocation of Mg as well as the partitioning of photoassimilates is maintained why these plants do not show a restricted biomass development, no chloroses or necrosis on leaves, no increased expression levels of Mg transporters, and no increased concentrations of other cations or ions in leaves.

All described relations between Mg status, photoassimilate partitioning (mainly via sucrose), Mg transport, concentrations of other cations and of ions, and finally biomass development of potato are schematically illustrated in Fig. 6.

## Supplementary information

Supplementary Information.
