# Author Correction: Root growth in light of changing magnesium distribution and transport between source and sink tissues in potato (*Solanum tuberosum* L.)

**DOI:** 10.1038/s41598-020-72313-y

**Published:** 2020-09-11

**Authors:** Mirjam Koch, Merle Katharina Winkelmann, Mario Hasler, Elke Pawelzik, Marcel Naumann

**Affiliations:** 1 Department of Crop Sciences, Division Quality of Plant Products, University of Göttingen, Carl-Sprengel-Weg 1, 37075 Göttingen, Germany; 2 Variationsstatistik, Christian-Albrechts-University of Kiel, 24098 Kiel, Germany; 3 Present Address: Albrecht Daniel Thaer-Institute of Agricultural and Horticultural Sciences, Crop Science, Humboldt-University of Berlin, Albrecht-Thaer-Weg 5, Berlin, Germany; 4 Present Address: Department of Agricultural Sciences and Landscape Architecture, University of Osnabrück, Albrechtstraße 30, 49076 Osnabrück, Germany

Correction to: *Scientific Reports* 10.1038/s41598-020-65896-z, published online 29 May 2020

The Acknowledgements section in this article is incomplete.

“We thank the K + S GmbH for the financial support and the Division of Agronomy, University of Göttingen for allowing us to use their devices – with special thanks to Juliane Streit. Besides, our thanks go to Arne Gull for performing the anion measurements.”

should read:

“We thank the K + S GmbH for the financial support and the Division of Agronomy, University of Göttingen for allowing us to use their devices – with special thanks to Juliane Streit. Besides, our thanks go to Arne Gull for performing the anion measurements. We acknowledge support by the Open Access Publication Funds of the Göttingen University.”

Additionally, there is an error in Figure  5B where the label ’12 DAO’ was incorrectly formatted. The correct Figure 5 appears below as Figure 1.

**Figure 1:**
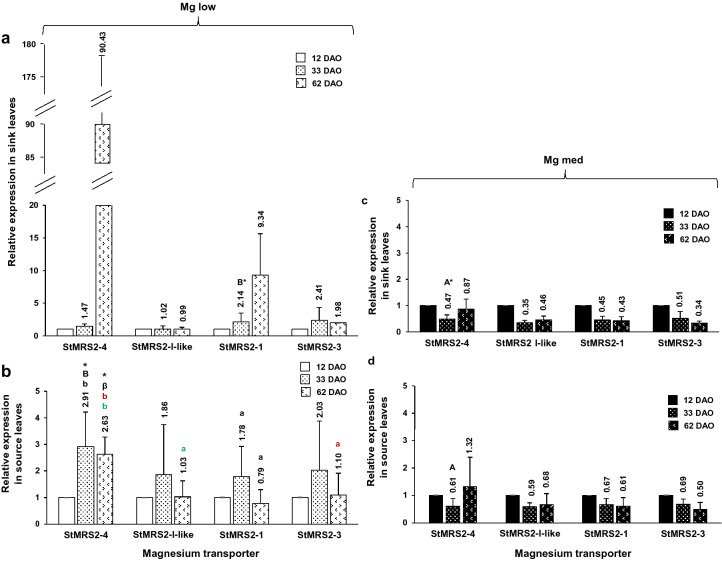
.

The Article also contains errors in Table 1 where the heading ‘Roots’ was incorrectly produced as a standard row. The correct Table 1 appears below.

**Table 1:** Mineral nutrient concentrations in mg g^−1^ DW in source leaves at 49 days after onset of the treatment (three days before root harvest) and in roots at harvest (55 days after onset of the treatment) (n = 4) (Experiment 1). ‘Mg low’ = 5 µM Mg; ‘Mg med’ = 100 µM Mg; ‘Mg high’ = 500 µM Mg. K = potassium, Ca = calcium, P = phosphorus, S = sulphur, B = boron, Fe = iron, Mn = manganese, Na = sodium, Zn = zinc. Mean ± SE values. Capitals = significant differences between ‘Mg low’, ‘Mg med’, and ‘Mg high’ plants. No indication = not significant. *P* < 0.05.

	Mg low		Mg med		Mg high	
**Leaves**						
K	66.88 ± 2.63	B	60.71 ± 5.38	AB	56.65 ± 4.44	A
Ca	12.32 ± 3.16		12.31 ± 1.03		14.04 ± 3.43	
P	6.12 ± 1.34		6.75 ± 1.63		4.79 ± 1.38	
S	3.13 ± 0.40	A	4.10 ± 0.52	B	3.52 ± 0.84	AB
B	0.05 ± 0.01		0.05 ± 0.01		0.05 ± 0.01	
Fe	0.10 ± 0.01	A	0.16 ± 0.03	B	0.11 ± 0.02	AB
Mn	0.06 ± 0.01	B	0.04 ± 0.01	A	0.05 ± 0.01	AB
Na	0.19 ± 0.04	B	0.09 ± 0.02	A	0.06 ± 0.02	A
Zn	0.06 ± 0.02		0.05 ± 0.02		0.03 ± 0.01	

**Roots**						
K	2.32 ± 1.27		1.71 ± 0.22		3.86 ± 2.58	
Ca	13.83 ± 2.27	AB	13.91 ± 1.18	B	11.33 ± 0.46	A
P	4.46 ± 0.94		3.86 ± 0.32		3.47 ± 1.09	
S	2.87 ± 0.52		3.11 ± 0.16		3.29 ± 0.32	
B	0.02 ± 0.01		0.02 ± 0.01		0.02 ± 0.01	
Fe	4.58 ± 0.41	B	2.36 ± 0.61	A	1.98 ± 0.86	A
Mn	0.33 ± 0.07	B	0.17 ± 0.02	A	0.14 ± 0.05	A
Na	0.35 ± 0.02		0.36 ± 0.03		0.37 ± 0.03	
Zn	0.18 ± 0.07	B	0.04 ± 0.01	A	0.04 ± 0.01	A

