# Supplementary material for: Root growth in light of changing magnesium distribution and transport between source and sink tissues in potato (Solanum tuberosum L.)
Source: Sci Rep. 2020 May 29;10:8796. doi: 10.1038/s41598-020-65896-z (PMC7260234; doi:10.1038/s41598-020-65896-z)
Supplement: Supplementary file 1 — Supplementary Information. [file 41598_2020_65896_MOESM1_ESM.docx]

**Root growth in light of changing magnesium distribution and transport between source and sink tissues in potato (*Solanum tuberosum* L.)**

Mirjam Koch^1,2^*,Merle Katharina Winkelmann^1,3^, Mario Hasler^4^, Elke Pawelzik^1^, and Marcel Naumann^1^

^1^Department of Crop Sciences, Division Quality of Plant Products, Carl-Sprengel-Weg 1, 37075 Göttingen, University of Göttingen, Germany.

^2^present address: Department of Crop and Animal Sciences, Division of Crop Science, 14195 Berlin, Humboldt-University of Berlin, Germany.

^3^present address: Department of Agricultural Sciences and Landscape Architecture, Albrechtstraße 30, 49076 Osnabrück, University of Osnabrück, Germany.

^4^Variationsstatistik, 24098 Kiel, Christian-Albrechts-University of Kiel, Germany.

*Correspondence to: Mirjam Koch, email: [mirjam.t.koch@hu-berlin.de](mailto:mirjam.t.koch@hu-berlin.de), phone number: 49(0)30-2093-46474.

**Supplementary Figure 1:** Mean temperature, precipitation, and irradiance over the vegetation period (outdoor installation, Experiment 2).

**Supplementary Table 1:** Soil status of soil used for plant propagation prior onset of the treatment without fertilization in Experiment 1. C_org_ = organic carbon, total N = total nitrogen, C/N ratio = carbon/nitrogen ratio, P = phosphorus, S = sulphur, K = potassium, Mg = magnesium, Cu = copper, Mn = manganese, B = boron, Zn = zinc, Na = sodium. All soil parameters were assessed according to standard procedures by LUFA Nord-West, Institute for Soil and Environment.

| Soil parameter | |  |
| --- | --- | --- |
| pH | 6.6 | |
| C_org_ (%) | 0.04 | |
| Total N (%) | < 0.01 | |
| C/N ratio | 4 | |
| Humus content (%) | 0.1 | |
| Mineral nutrients (mg 100 g^-1^ soil) | | |
| P | < 1 | |
| S | 5.95 | |
| K | 1.5 | |
| Mg | < 1 | |
| Cu | 0.1 | |
| Mn | 0.2 | |
| B | < 0.10 | |
| Zn | 1.6 | |
| Na | 6.45 | |

| Nutrient [Form of nutrient] | Applied amounts of nutrient  ‘Mg low’ and ‘Mg med’ plants | Applied amounts of nutrient  ‘Mg high’ plants |
| --- | --- | --- |
| Magnesium [Mg_2_SO_4_ • 7 H_2_O] | 5 | 100 |
| Potassium [K_2_SO_4_] | 300 | 300 |
| Nitrogen [Ca(NO)_3_] | 300 | 300 |
| Phosphorus [Ca(PO_4_)_2_ • H_2_O] | 100 | 100 |
| Calcium [CaCO_3_] | 1300 | 1300 |
| Boron [H_3_BO_3_] | 2 | 2 |
| Zinc [ZnSO_4_ • 7H_2_O] | 2 | 2 |
| Molybdenum [Na_2_MoO_4_ • 2 H_2_O] | 0.01 | 0.01 |
| Copper [CuSO_4_ • 5H_2_O] | 2 | 2 |
| Manganese [MnSO_4_ • H_2_O]  Iron [Fe(III) EDTA (13% Fe)] | 6  3 | 6  3 |

**Supplementary Table 2:** Used form and applied amounts (mg kg^-1^ soil) of nutrients for the fertilization of the soil (Supplementary Table 1) used for plant propagation before transfer into nutrient solution in Experiment 1. Plants, which were later treated in nutrient solution with 5 and 100µM Mg (‘Mg low’ and ‘Mg med’ plants), were applied with 5 mg Mg kg^-1^ soil. Plants, which were later treated in nutrient solution with 500µM Mg (‘Mg high’ plants), were applied with 100 mg Mg kg^-1^ soil.

**Supplementary Figure 2:** Schematic setups of Experiment 1 and 2. Experiment 1 had three levels of Mg supply (‘Mg low’ = 5 µM Mg; ‘Mg med’ = 100 µM Mg; ‘Mg high’ = 500 µM Mg). Experiment 2 had two levels of Mg supply (‘Mg low’ = 5 µM Mg; ‘Mg med’ = 100 µM).

**Supplementary Table 3:** Gene names, NCBI Reference Sequences, UniProtKB name, forward primer sequence, reverse primer sequence, and amplicon sizes (bp).

| **Gene** | **NCBI RefSeq** | **UniProtKB** | **forward primer** | **Reverse primer** | **Amplicon size** |
| --- | --- | --- | --- | --- | --- |
| **StMRS2-4** | XM_006349290 | M1CBU7 | TTGCTTGATCCTCTACGTCA | TCAAGCACCTGAAACTCAAA | 209 |
| **MRS2-I-like** | XM_006351568 | M1B7M8 | TGACAATGTTACTCCCGTTG | TCATGCTGAACATTGGAATC | 105 |
| **StMRS2-1** | XM_006356192 | M1CRS8 | GGTGCCTGACTTCTACGTTT | TACCTGTAGATCGGGAGAGC | 193 |
| **StMRS2-3** | XM_006346224 | M1CF18 | ACTGGACAGACCCAAGTTGT | GGCGGTAATAATAGCCTTGA | 174 |
| **StUbiquitin** | NM_001288394 | A9YTY9 | CACCAAGCCAAAGAAGATCA | TCAGCATTAGGGCACTCCTT | 120 |

**Supplementary Figure 3:** Effect of Mg deficiency on above and below ground biomass in Experiment 2 at harvest (78 days after onset of treatment). (a) shoot biomass (n = 11-15), (b) root biomass with shoot-to-root biomass ratios (mean ± SE values above bar plot) (n = 11-15), and (c) total root length (n = 3) and Mg root concentrations (n = 11-15) under low (‘Mg low’) and under medium (‘Mg med’) Mg supply. Mean ± SE values. *P* < 0.05; * = *P* < 0.01.

**Supplementary Figure 4:** Effect of Mg deficiency on plant height (a) and number of internodes (b) at 10 sampling dates under low (‘Mg low’) and under medium (‘Mg med’) Mg supply in Experiment 2 (n = 11-15). Mean ± SE values. Capitals = significant differences between ‘Mg low’ and ‘Mg med’ treated plants. No indication = not significant. *P* < 0.05; * = *P* < 0.01.

**Supplementary Figure 5:** Effect of Mg deficiency on soluble hexose sugar concentrations in sink and source leaves in Experiment 2. Hexose (fructose and glucose) sugar concentrations in sink (a) and source (b) leaves under low (‘Mg low’) and medium (‘Mg med’) Mg supply at days 40, 54, and 76 after onset of treatment (DAO) (n = 4). ‘Mg low’ = 5 µM Mg; ‘Mg med’ = 100 µM Mg. Mean ± SE values. No indication = not significant.
